# Power Law Distributions of Patents as Indicators of Innovation

**DOI:** 10.1371/journal.pone.0049501

**Published:** 2012-12-05

**Authors:** Dion R. J. O’Neale, Shaun C. Hendy

**Affiliations:** 1 Applied Mathematics Group, Industrial Research Limited, Lower Hutt, New Zealand; 2 MacDiarmid Institute for Advanced Materials and Nanotechnology, School of Chemical and Physical Sciences, Victoria University of Wellington, Wellington, New Zealand; Cinvestav-Merida, Mexico

## Abstract

The total number of patents produced by a country (or the number of patents produced per capita) is often used as an indicator for innovation. Here we present evidence that the distribution of patents amongst applicants within many countries is well-described by power laws with exponents that vary between 1.66 (Japan) and 2.37 (Poland). We suggest that this exponent is a useful new metric for studying innovation. Using simulations based on simple preferential attachment-type rules that generate power laws, we find we can explain some of the variation in exponents between countries, with countries that have larger numbers of patents per applicant generally exhibiting smaller exponents in both the simulated and actual data. Similarly we find that the exponents for most countries are inversely correlated with other indicators of innovation, such as research and development intensity or the ubiquity of export baskets. This suggests that in more advanced economies, which tend to have smaller values of the exponent, a greater proportion of the total number of patents are filed by large companies than in less advanced countries.

## Introduction

Endogenous theories of economic growth relate behaviour at the firm level to productivity growth through a variety of models of the innovation process [1], [2]. Despite the crucial role that firms and their inter-relationships must undoubtedly play in economic growth, a complete description of this complex system has eluded economists. More than a century ago, one of the fathers of modern economics, Alfred Marshall, drew on an analogy with forest ecosystems to describe this system: *“… we may read a lesson from the young trees of the forest as they struggle upwards through the benumbing shade of their older rivals. Many succumb on the way, and only a few survive… And as with the growth of trees, so it was with the growth of businesses…”* [3]. Today we have indications that this is more than just a metaphor. Many observations support the idea that, as with the distribution of biomass and metabolic rates amongst biological organisms [4], the distribution of firm sizes follows a power law [5]–[9].

The validity of theories of economic growth, endogeneous or otherwise, is generally judged by their ability to explain the variance in the rates of economic growth between different countries [10]. Although modern theories of growth often make use of firm level models of the innovation process, these theories are less often tested at this scale, despite our knowledge of a number of pertinent stylised facts. When aggregated at the city level, for instance, quantities such as the number of new patents, inventors, research establishments and even productivity have been found to follow power law scaling with respect to city size [11], [12]. Crucially, these measures of innovation scale super-linearly; larger cities are more productive per capita than smaller cities [13]. However when patent data is aggregated at the level of countries, rather than cities or regions, we find that larger countries do not consistently outperform smaller countries in innovation. Economic geography tells us that cities exist to exploit the benefits of agglomeration [14]–[16], so it is not necessarily surprising that super-linear scaling for cities can be seen in patent data. Countries exist for more complicated reasons.

Quantities such as patent counts or the number of researchers are only a proxy for innovation however. While the OECD regularly reports on national patent counts in its assessments of national innovation performance, it is often pointed out that the value of individual patents vary enormously [17]. This begs the question: are straightforward counts of patents a good indicator of innovation? Attempts to answer this by valuing patents have been made using patent citations [18] and through patent renewals [19]. In this article, we take another approach to measuring innovation by looking at the distribution of patents within an economy rather than just the total number of patents itself.

In particular, we report on several new stylised facts regarding the distribution of intellectual property amongst firms in national economic ecosystems. We consider the distribution of patents among applicants within countries and find compelling evidence that this distribution follows a power law in many instances. However these power laws are not universal: the best-fit exponents for these distributions differs from country to country by a statistically significant amount. This suggests that firms within the ecosystems of different countries experience different environments which influence their patenting behavior. Using a simple preferential attachment model, based on the Yule process, we show it is possible to reproduce the qualitative features observed in the empirical data and to explain some of the variation of exponents between countries. We also find that the value of the power law exponent is inversely correlated with a number of indicators that are commonly linked with innovation, such as research and development (R&D) intensity. Interestingly we find that the value of the exponent saturates at high R&D intensities.

## Methods

### Empirical Distributions of Patents

In this study we have used the Organisation for Economic Co-operation and Development (OECD) Harmonised Applicant Name (HAN) data set from July 2011. This includes patent applications filed at the European Patent Office (EPO) from 1977–2007 with partial data from 2007 onwards. The data set covers 22 countries and has been harmonised by the OECD to correct for cases where applicant details have been recorded differently on different patent applications. Details of the harmonization methodology are given in the documentation accompanying the data set [20]. The use of the harmonised data is important. When the same analysis was performed with non-harmonised data (from the OECD REGPAT data set), large errors resulted from fragmentation where a single unique applicant appeared as several different applicants due to, for example, variations in recording the applicant name. These errors are typically in the order of 5% to 15% of the value of the power law exponent, but are as high as 25% in cases.

Power law distributions are only one of many *right-skewed*, or *heavy-tailed* distributions. By power law distribution we mean one where the probability distribution 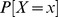 of a discrete variable 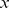 satisfies.
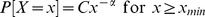
where 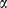 is called the power law exponent or scaling parameter. (Power law distributions are, of course, also defined for continuous variables. In fact much of the resulting analysis is simpler in the continuous case. See [21] for a good overview of power laws in empirical data.) The scaling constant 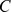 is determined by the requirement that probabilities sum to one: 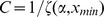, where 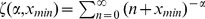 is the Hurwitz zeta function. To test rigorously whether the patent distributions observed follow power laws, we follow the procedure described in [21]. We use a maximum likelihood estimator for a discrete power law distribution to fit the exponent, and estimate the power law cut-off 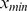 by choosing the value which minimises the Kolmogorov–Smirnov statistic, 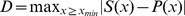, where 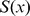 is the cumulative density function (CDF) of the data being fitted and 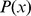 is the CDF of the fitted model distribution. The standard deviations of the fitted values were calculated using a boot-strapping method, drawing a sequence of points 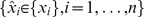 at random, uniformly, and with replacement from the original distribution. The fitted values are listed in Tab. 1 along with their estimated standard errors. For most data, a power law is only fitted to the tail of a distribution, i.e. for values greater than some 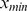. The patent distributions are interesting in that most countries show a good power law fit for the entire range of the data.

**Table 1:** Summary statistics for the patent distributions of the 22 countries.

ISO	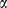	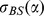	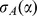	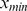	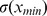	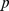	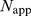	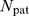
AT	1.97	0.074827	0.0044163	3	1.0777	0.01	3214	18398
BE	1.93	0.017827	0.0091749	1	0.1	0.24	2746	20470
CA	1.99	0.022143	0.0079035	1	0.3266	0.16	4842	24276
CH	1.86	0.036726	0.0028699	2	1.9159	0.01	7907	74987
CN	2.23	0.031152	0.020124	1	0.14071	0.81	1930	7879
CZ	2.36	0.12005	0.052896	1	0.25643	0.16	438	910
DE	1.97	0.059017	0.0010264	18	6.681	0.29	32558	391834
DK	2	0.031161	0.010195	1	0.39492	0.52	3039	15081
ES	2.13	0.020977	0.012197	1	0	0.14	3614	10408
FI	1.98	0.053119	0.005757	3	0.68895	0.36	2289	20378
FR	1.89	0.0096839	0.0032337	1	0.25643	0.45	18317	158608
GB	2.03	0.035597	0.0022348	3	0.98985	0.79	18041	99027
IE	2	0.029475	0.016786	1	0	0.63	1121	4074
IL	2.11	0.021996	0.012833	1	0	0.04	3019	9138
IT	2.21	0.0996	0.0025322	7	2.9426	0.99	14255	57260
JP	1.66	0.006795	0.0017311	1	0.17145	0	18121	508774
NL	1.97	0.012978	0.0062712	1	0.2	0.43	7043	79976
NO	2.12	0.027157	0.016935	1	0.1	0.28	1803	5742
PL	2.37	0.13149	0.049215	1	0.37753	0.15	523	985
PT	2.27	0.080284	0.054094	1	0.1	0.29	308	707
SE	2.03	0.015404	0.0077361	1	0	0.42	5998	35655
US	1.87	0.024768	0.00081815	7	2.2293	0.61	55539	654304

The best fit of the empirical data to a power law model is achieved with a power law exponent 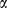 and cut-off 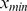. The estimated standard deviation in these parameters is also given. In the case of the standard deviation of 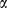, two estimates are given. The first, 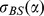, is calculated using a bootstrap method via the Matlab code which accompanies [21]. The estimate 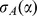 is obtained via the analytic expression eqn. (3.6) of [21] which gives an estimate for the standard error 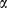, assuming that the underlying distribution is well fitted by a power law (i.e. the 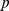-value is large). The estimate 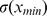 is calculated via the bootstrap method. The values for 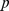 indicate the “goodness of fit” of the empirical data to a power law model. Also given, is 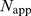 the number of applicants and 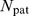 the total number of patents held.

In Tab. 1 we report two different estimates of the standard error in 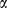, the first 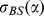 is estimated via bootstrap method using the Matlab code which accompanies [21]. This method can overestimate the value of the standard error in some cases – details are given in chapter six of [22]. The second estimate, 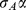 uses the analytic expression eqn (3.6) of [21] which gives an accurate estimate of the standard error for 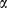, in the case where the underlying data are consistent with a power law model. When comparing the estimated power law exponents of empirical and simulated data, we use the boot strap estimate of the standard error in the value of 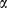 for the empirical data.

The values in Tab. 1 for the standard errors in 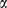 and 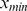 give us an indication of how precise the estimates of the best fit parameters are, but they do not tell us whether the power law model itself is a good fit. To quantify the goodness-of-fit of a power law model to the empirical data we calculate a so-called 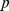 value [21]. The value of 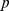 is essentially the fraction of the time when we might expect a goodness-of-fit as poor, or poorer than, that of the empirical data purely due to statistical fluctuations. A 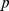 value of 1 would indicate that the amount of mis-fit between the data and a power law is entirely attributable to statistical fluctuations. Values of 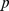 less than a threshold in the range of 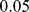 to 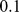 are typically used to rule out a power law fit. A threshold of 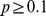 would exclude only four countries – Austria, Switzerland, Israel, and Japan.

### A Generative Model for Patent Distributions

We now describe a mathematical model which is intended to reproduce the behaviour observed in the empirical data. Since it is not clear why the distribution of patents amongst applicants should *necessarily* follow a power law, and since the underlying rules or patterns which lead to such a distribution for patents is not obvious, it is important that our generative model follows some set of rules or procedures which could credibly apply to growth in the number of patents.

There is a large literature on generative models for power law distributions, going back almost a century [23]. We use a model equivalent to the Yule process [24], based on two assumptions; 1) growth – the number of applicants with patents increases over time, and 2) preferential attachment – the likelihood of an existing applicant acquiring a new patent is proportional to the number of patents that the applicant already holds.

These assumptions lead to the following algorithm: Beginning with a single applicant holding a single patent, at each time step we either add a new applicant holding a single patent or add a new patent to an existing applicant. The rate at which new applicants are added is determined by the growth rate 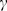, which is fixed throughout the simulation. When a patent is added to an existing applicant the probability that it is attached to applicant 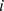 is given by 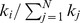, where 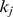 is the number of patents held by applicant 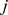 and 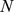 is the total number of applicants in the model at that time step. Since only a single patent is added at each time step, the (inverse of the) growth rate 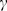 gives (for large 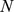) the average number of patents per applicant. Since it is desirable that a model should reproduce known quantities, such as the average number of patents per applicant, we choose 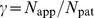 using the empirical data in Tab. 1. This ensures that this quantity matches that observed empirically, and eliminates the only free parameter in the model.

It is not difficult to prove that such an algorithm produces data with a power law tail when the number of steps taken becomes large [23] and that the power law exponent for the simulated distribution tends towards 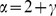 [25]. Hence, the exponents of the simulated distributions are bounded below by 2, approaching this limit as the average number of patents per applicant becomes large.

Using this algorithm we simulated the growth of the corresponding distribution of patents 500 times for each country, determining the average value of 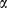 and its corresponding standard deviation. In addition to comparing the simulated results with the empirical patent data, we also tested the preferential attachment model against a simple null model where the preferential attachment rule was replaced by uniform random attachment.

## Results and Analysis

### Empirical Distributions of Patents

**Figure 1:**
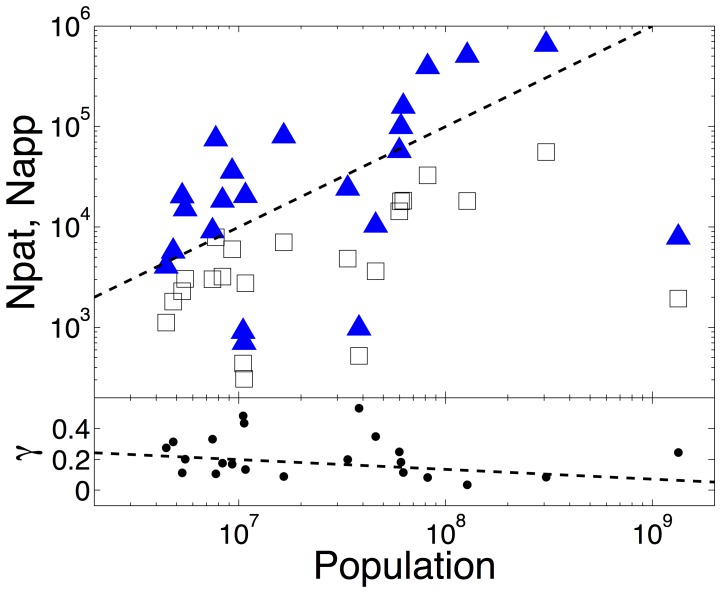
Scaling of number of patents and patent applicants, with country size. Upper plot: Number of EPO (European Patent Office) patent applications (filled triangles) and unique applicants (open squares) versus national population, for the 22 countries in the OECD HAN data set. The dashed line indicates the slope which the data would follow if they scaled linearly – in the absence of agglomeration effects. Lower plot: Ratio of number of applicants to number of patents (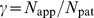) for the same data. The least squares best fit has a slope of 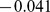 and 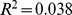 indicating a poor correlation and little dependence on population.

Several previous studies have considered patent data aggregated at the level of cities or metropolitan regions. In the method described above, we aggregate the patent data at the country level in order to better understand the relationship between national economies and innovation. In contrast to the findings for cities in [11]–[13] where data are tightly clustered and show clear super-linear scaling due to agglomoration effects, the number of patents per country varies roughly linearly with a country’s population and is poorly correlated in comparison with the results for cities, Fig. 1. We infer from this that the agglomoration effects observed for cities are absent at a national level. If we use patenting as a proxy for innovation, then we conclude that the drivers of innovation at a national level are different from those which lead to agglomeration effects for innovation within cities and regions.

**Figure 2:**
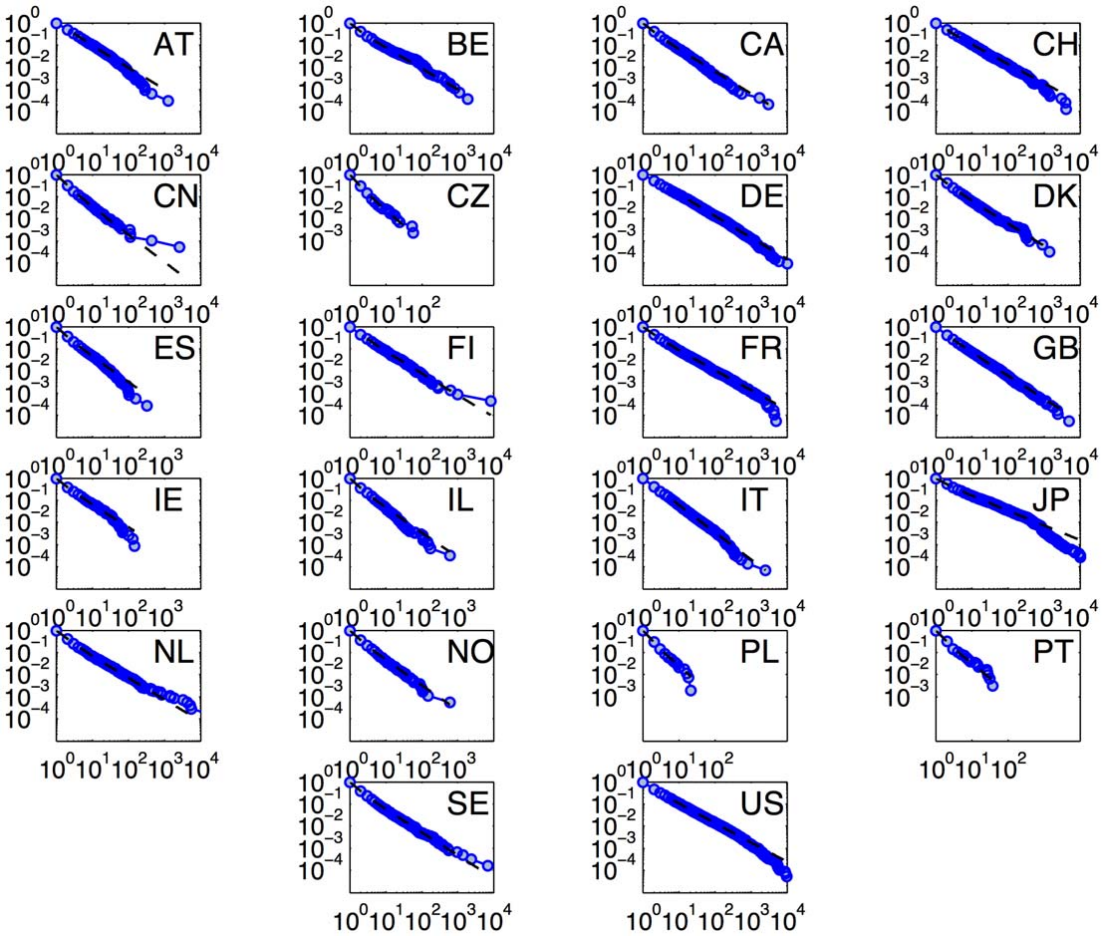
Cumulative density functions and power law fits for the 22 countries in the data set. The CDFs of EPO patent distributions for all 22 countries, ordered by country code, are indicated by blue circles. The slope of the best-fit power law model is shown as a black dotted line. The match to a power law fit is generally good.

As a first step towards investigating the distribution of patents amongst applicants, the cumulative density functions were plotted for the 22 countries. These are shown, along with the fitted power laws, in Fig. 2. The fit is generally strong, with only small deviations between the data and the fitted models over four orders of magnitude for most countries.

Various statistics such as the best-fit exponent, the 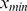 threshold of the power law tail and the goodness-of-fit 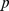 value were calculated from these distributions, and are presented in Tab. 1 The main feature of Tab. 1 is that the power law exponents for the 22 countries differ by an amount greater than their estimated standard deviations. The exponents mostly lie between 2 and 2.5, with the exponent for many countries being close to 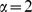 – the threshold below which the mean value of the distribution diverges. For countries with 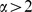, the expected mean value for the fitted power law distribution is given by 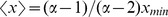, hence the expected mean number of patent applications per firm is large for many of the countries.

**Figure 3:**
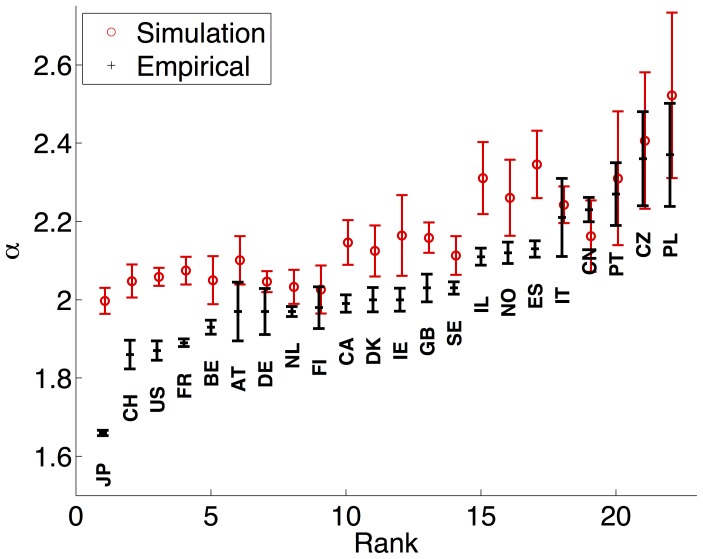
Power law exponents, sorted by rank. Values of 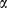, with their associated estimated uncertainties, for the 22 countries in the EPO HAN data set (black), sorted by 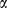, along with average 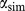 values (red), and their associated standard deviations, for simulated data. For each country’s simulation, the growth rate was determined by the ratio 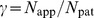 from Tab. 1.

**Figure 4:**
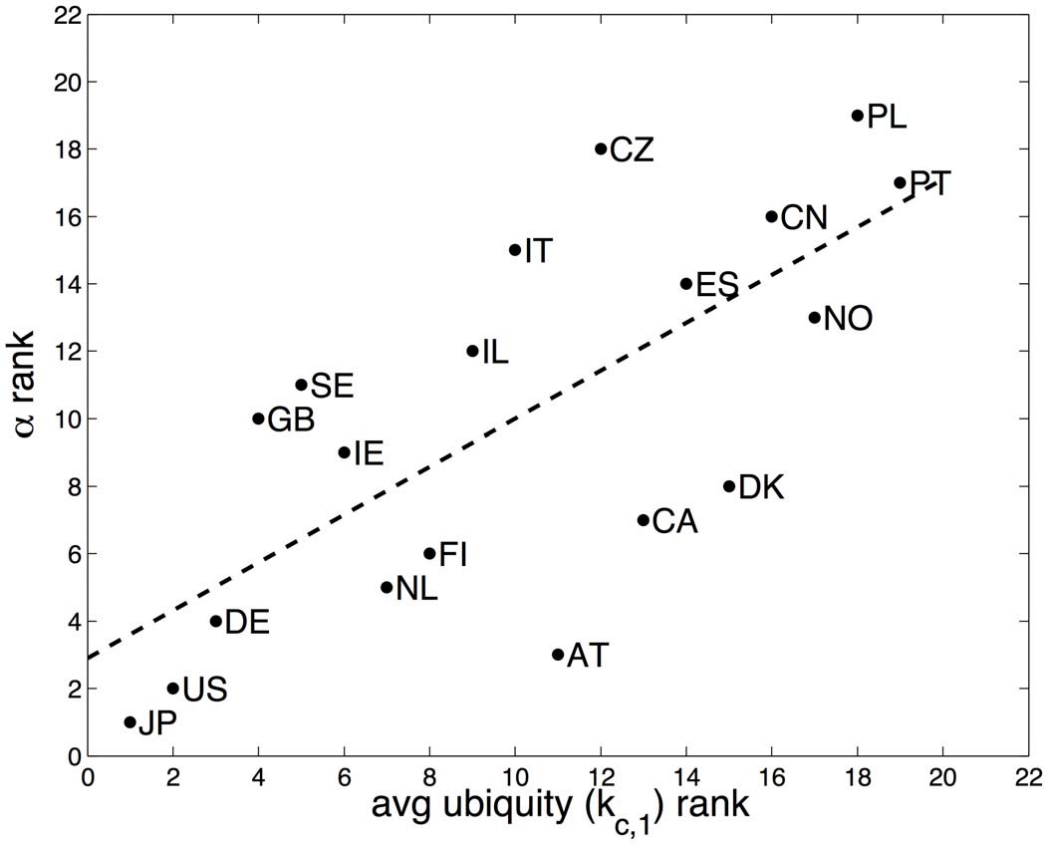
Power law exponents are correlated with the “ubiquity” of exported products. Correlation between the country rank of the empirical power law exponent 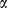 and the country rank based on average export ubiquity (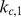 as reported in [26]). The dashed line indicates the linear least-squares fit and has slope 0.71 (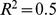, 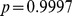).

Figure 3 shows a plot of the power law exponents and their estimated standard deviations for each country. We order the countries by the size of the exponent 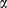. It is interesting to note that the countries which are often thought of as having innovative or “high-tech” economies lie mostly towards the left of the plot, with smaller power law exponents. The link between small exponents and economies with highly specialized firms is reinforced by Fig. 4 where the rank of the countries by exponent is plotted against the rank for the average “ubiquity” of the goods exported by that country [26]. A low ubiquity rank indicates that the goods exported by a country are exported by few other countries. Hidalgo and Hausmann observed in the supplementary material of [26] that industrialized countries export products in almost *all* product categories, hence, specialization patterns are empirically driven by the *lack* of diversification amongst less developed countries. We therefore use the low ubiquity rank of a country as an indicator of specialized and complex exports. The correlation between the exponent rank and ubiquity rank indicates that the power law exponents give information about the presence of sophisticated (export) sectors in a country. The lower the value of the power law exponent of a country, the more likely that the country exports a number of specialised goods, exported by few other countries.

The data used for the empirical analysis in this section is limited to patents filed at the European Patent Office. However, the 22 countries included in this data extend beyond European countries, which raises the possibility of systematic regional biases within the data; the considerations and behaviour of an applicant filing at a local patent office may differ from an applicant who is pursuing protection at a foreign office. To determine that regional effects do not invalidate our results we have repeated this analysis for patents applications filed under the Patent Cooperations Treaty (PCT). The PCT provides a unified procedure for filing patent applications and establishing precedence under international law. Since PCT patent applications are equivalent for all of the contracting states of the treaty, and since the treaty covers most industrialised nations, PCT applications can be expected to be free of any regional bias. The PCT patent records are also drawn from the HAN data set and hence use the same applicant harmonisation method and cover the same timeframe as the EPO records. As with the EPO applications, patent distributions for PCT applications aggregated at national level are well described by a power law with an exponent that closely matches those from the EPO data.

**Figure 5:**
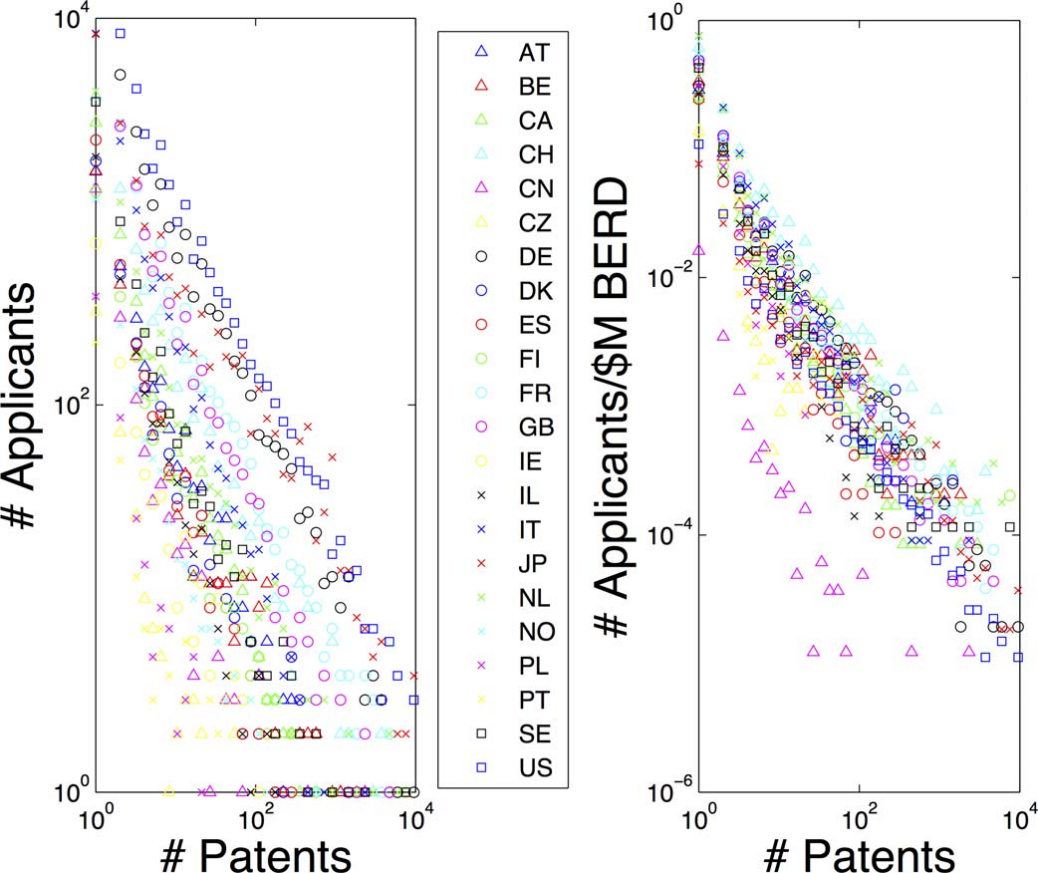
Expenditure on R&D rescales national patent distributions. Just as the total biomass of an ecosystem can be used to rescale the distribution for the frequency of organisms with a particular body mass in some area, total expenditure on R&D in a country can be used to rescale the distribution for the frequency of applicants in a country who have filed a particular number of patents. The left-hand plot shows the unscaled data, with each country indicated by a different color/shape of symbol. The right-hand plot shows the same data after the frequencies are scaled by business expenditure on R&D (millions of US dollars). The role played by GERD (gross expenditure on R&D) is similar. The one country for which the distribution does not match the others after rescaling (pink triangles) is China; a country which has only a recent record of filing patents at the EPO.

The well known correlations that exist between patent counts and R&D expenditure suggest that there may be a relationship between the patent distributions and national expenditures on R&D. Just as the total biomass of a natural ecosystem can be used to normalise frequency versus body mass distributions, rescaling the absolute number of patents for a country by that country’s absolute GERD or BERD causes the patent distributions to collapse on one another (see Fig. 5). Thus, in an innovation ecosystem, gross expenditure on research and development (GERD) and business expenditure on research and development (BERD) could be considered to play a role similar to that played by biomass in natural ecosystems [27].

**Figure 6:**
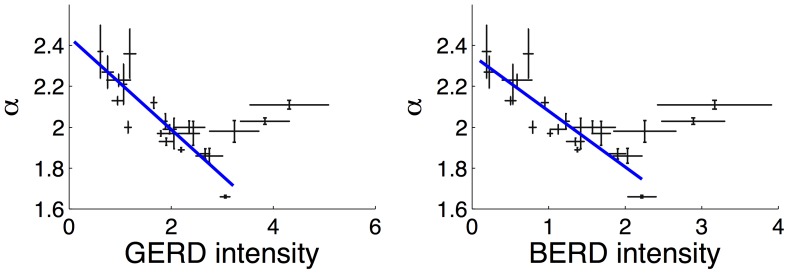
Lower values of the exponent 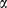 are inversely correlated with R&D spending. Power law exponents for EPO patent distributions of 22 OECD countries versus GERD (left) and BERD (right) intensity – gross, resp., business expenditure on R&D as a percentage of GDP. Vertical bars indicate the estimated standard error in the 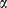 values, horizontal bars indicate the standard deviation in the time averaged (1995–2006) OECD data. The trend appears to be bimodal. For GERD, resp. BERD, intensity below approximatley 3% resp. 2% there is an inverse correlation between the power law exponent and the intensity of R&D spending. Beyond this level, the trend appears to reverse, though data in this region are limited. The blue lines indicate the least-squares linear regression fit to the data, excluding the three right-most points corresponding to Finland, Sweden and Israel (left to right). The linear fits are 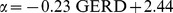 (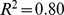, 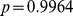) and 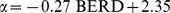 (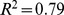, 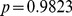).

It is also interesting to see whether the exponent 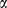 is related to expenditure on R&D. To investigate this, we plot the EPO power law exponents for the countries in the OECD HAN data set against GERD and BERD intensity as a percentage of gross domestic product (GDP): Fig. 6. We see a strong correlation between increasing intensity of expenditure on R&D, and lower values of the power law exponent (corresponding to more innovative and more sophisticated economies). An interesting feature is that the decrease in 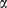 appears to saturate at about 3% GERD intensity, or 2% BERD intensity. Beyond this level of R&D expenditure, there is no evidence of further flattening of the patent distributions. It is also interesting to note that the correlation of 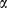 with both GERD and BERD is the same – a translation of BERD intensity by around 1% almost exactly matches the pattern for GERD intensity, implying that both BERD and GERD play similar roles.

### Generative Model

**Figure 7:**
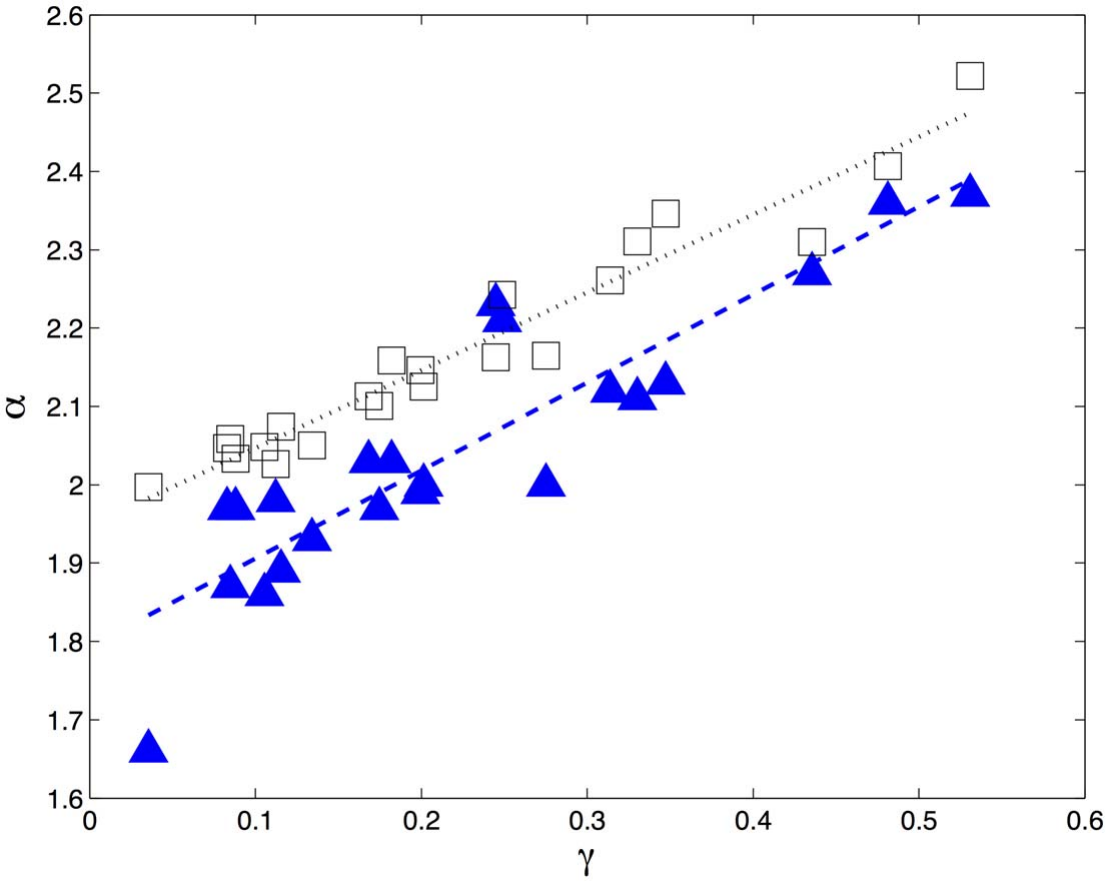
Power law exponents are inversely correlated with the average number of patents per applicant. Best fit power law exponents 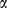 calculated for the empirical (filled triangles) and simulated (empty squares) patent distributions of each country as a function of 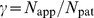. The linear least square fits are, respectively, 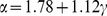 (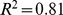, 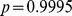) and 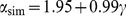 (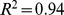, 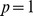).

The analysis used for fitting a power law to the empirical patent distribution was performed for each simulation run of the generative model. Figure 3 shows the values of 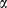 and the estimated standard errors for both the simulated and empirical data sets, again ordered by 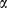 (for the empirical data). While the match between the empirical and simulated results is far from perfect, there is a clear qualitative fit: countries with lower values of 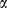 in the empirical data, show the same pattern in the simulated data. In Fig. 7 we show the relationship between the exponent 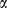 and the growth rate 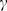 for empirical and simulated data. Both the empirical and simulated data show a clear correlation with 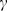, although the empirical data consistently has lower exponents than the simulated data. The linear regression fit for the simulated data is very close to the asymptotically expected result: 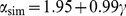, 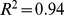, 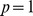, so the gap between the simulated and empirical exponents is not due to the finite duration of the simulations.

The distributions resulting from the null model, where the preferential attachment rule was replaced by uniform random attachment, are poorly fitted by a power law distribution: if a power law fit is assumed then the resulting best fit exponent is typically between four and six (c.f. 1.66 and 2.37 for the empirical data), shows no particular correlation with the growth rate 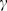 (a linear least-squares fit gives 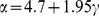, 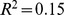, c.f. 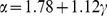, 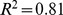, 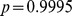 for the empirical data). Furthermore, the estimated standard deviation in the fitted power law exponents was typically greater than the total variation between exponents for the empirical data.

The relatively good agreement between the simulated and empirical data gives support to the assumption that applicants holding many patents are more likely to acquire further patents – future innovators are likely to also be past innovators. However we note that in our model, applicants can continue to acquire patents indefinitely. Clearly, this assumption is not realistic – applicant firms can go out of business or be acquired by other firms. Similarly, a single patent may be owned by more than one applicant, resulting in a network of co-applicants, (although 88% of the patents in the HAN EPO data set have only a single applicant). Both these effects will alter the patent distribution from that seen in the simulations.

## Discussion and Conclusions

In summary, we have found that the distribution of patents amongst applicants within OECD countries generally follows a power law, and that these power laws are not universal in that their scaling exponents differ significantly between countries. Using this exponent as a proxy for innovation provides a new way of looking at the structure of national economies and strengthens the analogy between innovating firms and ecosystems.

There have been many studies that have linked the market value or productivity of firms to their stock of knowledge as measured by R&D expenditure and patents held [28]. This suggests that the distributions of productivity and patents are also linked at the firm level. It is remarkable then that the characteristics of the overall patent distributions found here vary so little from country to country, despite the variety of sizes, locations and industrial structures encompassed by this set of countries. In fact it appears that much of the difference between countries can be captured by rather simple measures such economy-wide research and development expenditures and intensities.

This finding adds to a body of stylised facts concerning the distribution of the revenues and productivity of firms. For instance, it was observed some time ago that the tail of the distribution of the revenue of firms is well approximated by a power law [5]. Such a distribution can be reproduced by a model in which incumbant firms can innovate to improve their productivity but face competition from new entrants who are able to take advantage of existing technologies [8]. It has also been suggested that the distribution of the value of ideas may similarly have a power law tail [29], and processes whereby innovators select and improve on previously productive ideas have been shown to generate such distributions [30]. The variations between countries in the exponent of the power law tails seen here will provide an important test for future models of innovation by firms, and ultimately, long-run economic growth.

We note that there is certainly scope for increasing the sophistication of the generative model used here. For instance, it would be possible to modify this model by introducing a “death rate” for example, where applicants can cease to acquire new patents, and this would certainly change the resulting power law exponent. Such an approach would introduce an additional parameter to the model, which could be used to generate distributions which fit the empirical data much more closely. However, since there is no simple way to choose the death rate parameter *a priori* from the empirical patent data, such a model does not give additional insight into the process of innovation.

Similarly, the Yule process can be modified such that some patents are shared between applicants, resulting in a network of co-applicants. In this case, the choice of co-applicant for shared patents may also be determined by preferential attachment, leading to a network of applicants where the number of co-applicants per applicant also follows a power law distribution. Such models have been widely used, for example to model the distribution of links between pages on the world-wide-web [23].

Further modifications to the Yule process include the generalization proposed by Simon in [31] where preferential attachment is used to choose a *class* of applicants (i.e. those holding 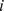 patents) but where the probability of obtaining within that class of an applicant obtaining a patent may be non-uniform. For example, it may depend on the amount of time since applicants last acquired a patent. It is also possible to modify the generative model such that the growth rate 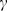 is not fixed but may depend on factors such as the number of patents already introduced to the model. Other variations of the Yule process such as nested Yule models can also be considered [32].

Finally, we remark on the fact that the power law exponents that describe these distributions are correlated with measures such as national expenditure on R&D, and the ubiquity, or degree of specialisation, of the basket of goods that a country exports. Countries that export more specialised goods tend to have a smaller proportion of companies that hold a larger share of the patents, while countries that export more ubiquitous goods tend to have a larger share of patents held in small portfolios. In Finland for instance, 80% of patents are owned by the top 10% of applicants, whereas in Portugal, only 50% of patents are owned by the top 10% of the applicants. Finland’s high R&D intensity and the low ubiquity of its exports suggest that Finnish firms are operating closer to the technological frontier than those of Portugal. The more highly skewed patent distribution that exists in Finland might indicate that new firms face higher barriers to entry, possibly because research and development in these countries takes place closer to the frontier. Thus it seems that the innovator of today is more likely to work in the research laboratory of a large multinational company than in the suburban garage or small start-up company.
